# Insulin Receptor Isoforms in Cancer

**DOI:** 10.3390/ijms19113615

**Published:** 2018-11-16

**Authors:** Veronica Vella, Agostino Milluzzo, Nunzio Massimo Scalisi, Paolo Vigneri, Laura Sciacca

**Affiliations:** 1 Department of Clinical and Experimental Medicine, Endocrinology Section, University of Catania Medical School, Garibaldi-Nesima Hospital, via Palermo 636, 95122 Catania, Italy; vellave@hotmail.com (V.V.); agomil@alice.it (A.M.); m.scalisi@hotmail.it (N.M.S.); 2 School of Human and Social Science, University “Kore” of Enna, 94100 Enna, Italy; 3 Department of Clinical and Experimental Medicine, University of Catania Medical School, Center of Experimental Oncology and Hematology, A.O.U. Policlinico Vittorio Emanuele, via Santa Sofia, 78, 95123 Catania, Italy; vigneri.p@unict.it

**Keywords:** insulin receptor isoforms, insulin receptor, IGF-1R, hybrid receptors, insulin, IGF-2, cancer, splicing factors

## Abstract

The insulin receptor (IR) mediates both metabolic and mitogenic effects especially when overexpressed or in clinical conditions with compensatory hyperinsulinemia, due to the metabolic pathway resistance, as obesity diabetes. In many cancers, IR is overexpressed preferentially as IR-A isoform, derived by alternative splicing of exon 11. The IR-A overexpression, and the increased IR-A:IR-B ratio, are mechanisms that promote the mitogenic response of cancer cells to insulin and IGF-2, which is produced locally by both epithelial and stromal cancer cells. In cancer IR-A, isoform predominance may occur for dysregulation at both mRNA transcription and post-transcription levels, including splicing factors, non-coding RNAs and protein degradation. The mechanisms that regulate IR isoform expression are complex and not fully understood. The IR isoform overexpression may play a role in cancer cell stemness, in tumor progression and in resistance to target therapies. From a clinical point of view, the IR-A overexpression in cancer may be a determinant factor for the resistance to IGF-1R target therapies for this issue. IR isoform expression in cancers may have the meaning of a predictive biomarker and co-targeting IGF-1R and IR-A may represent a new more efficacious treatment strategy.

## 1. Introduction

The insulin receptor (IR), a tyrosine kinase protein physiologically present in all mammalian tissues, is a heterotetramer composed of two extracellular α-subunits and two transmembrane β-subunits bound together by disulfide bonds [1,2].

The α-subunits present two different binding sites for the ligands, site 1 (low affinity-site) and site 2 (high affinity-site) [3,4,5,6]. Insulin first binds to the low affinity-site on one α-subunit and subsequently to the high affinity-site of the other α-subunit [4,7]; a second molecule of insulin binds to both sites of the α-subunits causing the dissociation of the first molecule [4]. After the binding of the ligands to the α-subunits, the tyrosine kinase activity of the β-subunits is triggered and then a cascade of intracellular proteins involved in cell metabolism, survival and growth are phosphorylated [8].

The main target tissues of insulin are liver, adipose tissue, skeletal muscle and brain, but IRs are ubiquitous and thus are expressed also in the heart, lung, pancreas, kidney, placenta, vascular endothelium, monocytes, granulocytes, fibroblasts and erythrocytes [9].

The IR and the insulin-like growth factor 1 receptor (IGF-1R) derive from a common ancestral gene, highly preserved in vertebrates and invertebrates [10] and show 45–65% homology in the ligand binding site and 60–85% homology in the tyrosine kinase and substrate recruitment domains [11,12]. Both receptors bind insulin, Insulin-like Growth Factors 1 (IGF-1) and Insulin-like Growth Factors 2 (IGF-2) although with different affinity.

The IR is encoded by the *INSR* gene, which is located in chromosome 19p13.2; the coding region includes 22 exons that generate a protein of 1370 amino acids, with a molecular mass of ≈154 Da [13]. Alternative splicing of exon 11 generates two structurally different isoforms: IR-A and IR-B. The 12 amino acids derived from exon 11 are included in the IR-B isoform (mature isoform), but not in the IR-A isoform (fetal isoform) [14,15]. The two isoforms are expressed differently, IR-A is predominantly expressed in embryo and fetal tissues, central nervous system (CNS), hematopoietic cells and cancer cells, whereas IR-B is mainly expressed in the major insulin target tissues, i.e., liver, fat and muscle [13,14,16,17]. 

**Figure 1:**
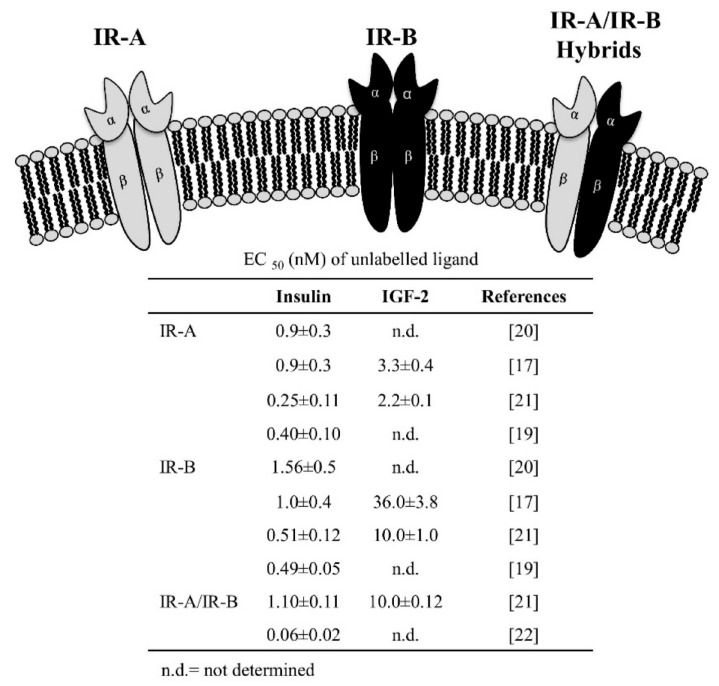
Schematic representation of the insulin receptor (IR) isoforms. Homodimerization of the αβ subunits A or B produces IR-A and IR-B. Heterodimerization of one A and one B αβ subunit forms the hybrid IR. The isoforms are generated by alternative splicing of exon 11 that encoded a 12-amino acid segment in the C-terminus of the α subunit, present in IR-B, but not in IR-A and this difference cause the difference in structure/function. The table indicates the affinity binding (expressed as EC_50_ of unlabeled ligand) to insulin and IGF-2 for the IR isoforms and for IR-A/IR-B.

IR-B binds insulin with high affinity and IGF-1 and IGF-2 with low affinity (Figure 1); IR-A binds with high affinity insulin and IGF-2, whereas it binds IGF-1 with approximately tenfold lower affinity [13,17,18,19,20] (Figure 1).

When activated by insulin, the IR-B mediates mainly metabolic effects, whereas the IR-A, activated by insulin or IGF-2, mediates mitogenic effects more than IR-B [14]. Both stromal and epithelial cancer cells can locally produce IGF-2 and, since IR-A is a high affinity receptor for IGF-2, the autocrine/paracrine IGF-2/IR-A loop promotes cancer cell proliferation.

IR isoforms are expressed as both homodimers (IR-A/IR-A, IR-B/IR-B) and heterodimers (IR-A/IR-B hybrid). In addition, IR isoforms may also form heterodimers with IGF-1R (IR-A/IGF-1R, IR-B/IGF-1R hybrid receptors) [21,22]. The binding affinity of hybrid receptors for the three ligands are not well established because only a few studies are available and they have used different methods [21,22] (Figure 1).

The IR is overexpressed in several malignancies determining abnormal responses to insulin, proinsulin and insulin-like growth factors, with predominant mitogenic rather than metabolic effects in malignant cells [8,17,23,24]. The biological role of the overexpressed IR in cancers is not yet well understood. A better knowledge may have important implications, considering that in clinical trials the target therapy with inhibition of IGF-1R is often inadequate to inhibit cancer progression [25], probably because the mitogenic signal might be propagated by the IR, an alternative pathway to the inhibited IGF-1R. Several studies have highlighted the role of the abnormal expression of IR isoforms in cell proliferation and cancer [14,16,17,26]. Many malignant cells, in fact, in addition to IR overexpression, predominantly express the IR-A isoform with an increased IR-A:IR-B ratio [13,27]. The abnormal IR transcript splicing causing an increased IR-A:IR-B ratio is a mechanism that potentiates the mitogenic response of cancer cells to circulating insulin and IGFs favoring tumor growth and progression [13]. In certain cancer types, the altered IR isoform expression may play a more important role than the IGF-1R itself.

In this review, we will summarize general data and the very recent studies on the role of the IR isoforms in many cancer types, and the possible mechanisms favoring the overexpression of IR-A isoform in malignant cells.

## 2. Deregulation of IR in Cancer

Numerous in vitro and in vivo studies demonstrated the role of IR and its isoforms deregulation on cancer development and progression. The IR deregulation occurs through two main mechanisms in cancer cells: Receptor protein overexpression and predominant A-isoform expression.

### 2.1. IR Overexpression

Several studies documented IR overexpression in a number of malignant cells (Table 1).

We first reported more than two decades ago that IR content is significantly increased in human breast cancer specimens compared to normal breast tissues: The mean value in cancer was more than six fold higher than normal breast tissue and only approximately 20% of cancer tissues had IR content in the normal range [28]. IR overexpression correlated with tumor size, cancer staging and estrogen receptor (ER) expression [28]. In addition, in node-negative breast cancer specimens, the IR content was a major predictor of reduced disease free survival (DFS) [49]. In the following years, many studies confirmed this observation, which is considered, together with hyperinsulinemia, the reason for increased breast cancer in obese and diabetic women. Recently, a specific role of IR has been hypothesized in breast cancer progression through a novel mechanism involving CD24, a common cell surface marker for breast cancer stem cells, linked to IR for promoting tumor growth [50].

Liver cancer, with a two-fold increased relative risk, is the most increased cancer in diabetic patients [51]. The Insulin/IR pathway is a major regulator of liver cells growth. The endogenous insulin, released by pancreatic cells in the portal system, reaches the liver at a concentration higher than peripheral tissues, because at first pass a relevant insulin aliquot (20–40%) is degraded.

The overexpression of the IR and the associated glycogen accumulation mediated by the IRS-1 and IRS-2 pathways, have been observed in an early phase of hepatocarcinogenesis in a rat model of liver cancer induced by genotoxic *N*-nitrosomorpholine exposition [40].

The IR overexpression is also involved in prostate cancer biology. IR is significantly overexpressed in human prostate cancer tissue relative to benign prostate tissue [33]. In a recent study, which included 130 men who underwent prostatic surgery, a significant increase of IR expression was found in formalin fixed paraffin embedded (FFPE) malignant specimens. A role of the IR in prostate cancer recurrence was not identified, since IR expression was similar in subjects with or without biochemical cancer recurrence [32].

A recent study in bladder cancer compared IR expression in blood vascular endothelial cells from human invasive bladder cancers and normal bladder tissues [42]. The IR resulted overexpressed in blood vascular cells of invasive bladder cancer and its expression was significantly related with a shorter progression-free and overall survival, suggesting a role of the IR as a predictor of cancer progression.

A number of studies investigated the IR in lung cancer. In a large series of 614 non-small cell lung cancer (NSCLC) the IR-A:IR-B ratio was significantly increased compared to 92 control normal lung specimens. In addition, 11% of the NSCLC specimens expressed only the IR-A isoform [43]. Moreover, in a strongly oncogenic mouse model (K-Ras activation and p53 loss) the inhibition of the IR signaling pathway, through the block of IRS-1 and IRS-2, suppressed cancer initiation in lung cells and improved the mouse survival [52]. Finally, in non-small cell lung cancer cell IR-knock down caused a strong induction of pro-apoptotic cytokines (IL-20, TNF-α), suggesting a role of IR in tumor cell survival via suppression of pro-apoptotic cytokines [53].

An increased IR expression was also found in thyroid cancer [45,54]. In particular, IR expression was increased both in differentiated thyroid carcinomas and also in benign non-functioning (“cold”) thyroid nodules, but not in highly differentiated hyperfunctioning adenomas. These findings suggest that IR overexpression may play a role in the early tumorigenesis of thyroid cancer [45]. 

Finally, in the atypical teratoid/rhabdoid tumor, a highly aggressive pediatric cancer of the central nervous system, a peculiar autocrine signaling involving insulin and the IR was discovered, modulating the tumor growth. Atypical teratoid/rhabdoid cancer cells overexpress the IR and produce insulin, activating an autocrine IR/PI3K/Akt/mTOR loop biologically relevant for cell proliferation [55]. 

In conclusion, abundant data in the literature indicate that IR overexpression is a characteristic of many cancers in different tissues and this observation is confirmed when IR content is measured in cancer and adjacent normal tissue from the same patients [17]. 

### 2.2. IR Isoform Expression

In breast cancer, IGF-2 is secreted by both epithelial and stromal cells and binds and activate both IR and IGF-1R. IR-A overexpression in breast malignant cells potentiates the mitogenic effects, because the high binding affinity of this IR isoforms for IGF-2 [2,29]. In breast cancer cells, blocking either IGF-2 or the IR markedly inhibited growth, demonstrating the relevance of the IR-A/IGF-2 loop in cell growth [29]. To fully inhibit the biological effects of autocrine IGF-2, both IR-A and IGF-1R need to be blocked. IR-A levels, measured by highly sensitive q-PCR in FFPE tissues of ER+ breast cancers and ER+ hormone refractory breast cancers (HR ER+) (i.e., tumors resistant to hormonal therapies), were greater than in ER− tumors [56]. Moreover, IR-A content in HR ER+ cancers was higher than IGF-1R content. In addition, an increased IR-A mRNA expression with a decreased IR-B expression results in an increased IR-A:IR-B ratio, as was detected in human breast cancer (Table 1) [30,31]. The IR isoforms expression is different in distinct breast cancer ER+ subtypes that have a different response to hormone treatment and a different prognosis.

The IR-A:IR-B ratio is significantly higher in the luminal B subtype characterized by tamoxifen resistance and a more unfavorable prognosis than in the luminal A breast cancer histotype characterized by response to hormonal therapy and relatively good prognosis [31].

This evidence supports the possibility that IRs dysregulation of IR isoform expression with an increased IR-A:IR-B ratio are related to more aggressive and prognostically unfavorable breast cancers. Therefore, the expression of IR isoforms in breast cancers may be used as a predictive biomarker for IGF targeted therapy [56,57].

Several in vitro and in vivo models’ studies confirm IR-A overexpression also in prostate cancer cells [58]. Both insulin and IGF-2 ligands participate to the detrimental effect of IR-A predominance [35]. Prostate cancer cell exposure to IGF-2 results in a reduced IR-B expression and the relative prevalence of IR-A [35] and IR-A:IR-B ratio is significantly increased in primary human prostate cancers compared to benign tissues [34].

IR-A was overexpressed during the normal proliferative phase (20-fold more than IR-B) in 87 women with either normal, or hyperplastic or neoplastic endometrium, suggesting that IR-A overexpression may be a mechanism involved in estrogen-independent endometrial proliferation [37]. In women with normal glucose tolerance or type 2 diabetes, IR-A was overexpressed in 75.7% endometrial cancer specimens compared to 35% of normal endometrial specimens and IR-A activation was documented to promote cancer cell growth [36]. The IR-A:total IR ratio was significantly higher in endometrial cancer compared to normal specimens, especially in women with type 2 diabetes (Table 1) [36].

Moreover, few available data indicate a predominant IR-A expression in ovarian cancer suggesting a possible role of this IR isoform in the regulation of ovarian cancer cell growth [38].

The overexpression of IR-A and the increased IR-A:IR-B ratio has been reported as a possible mechanism responsible for the adverse effects of hyperinsulinemia in a panel of 85 human hepatocellular carcinomas (Table 1) [39]. Recent data obtained in a model of liver cancer induced by diethylnitrosamine, confirm the role of the IR-A/IRS-1 system deregulation in this neoplasia [41].

In non-small cell lung cancer (NSCLC) specimens the mRNA expression of IR-B was significantly lower in cancer tissue compared to normal tissue, with an increase of the IR-A:IR-B ratio [43].

It has been hypothesized that NSCLC cancers may have both metabolic and mitogenic advantages from the increased IR-A:IR-B ratio, and therefore an evaluation of the IR isoforms before starting a treatment targeting the IGF pathway could be helpful [43].

IR isoform deregulation influences the biology of colon cancer and of colon pre-malignant lesions. The risk of adenoma is increased in subjects with hyperinsulinemia, hyperglycemia and increased c-peptide [59,60]. This risk appears related to a reduced apoptosis of normal rectal mucosa in these subjects and may suggest a role of insulin in the early-phase of the adenoma-carcinoma initiation process.

The differential expression of IR isoforms in stem cells, cancer cells and differentiated intestinal epithelial cells was evaluated in a mouse model of precancerous adenoma and in wild-type mice [44]. IR-A was the dominant isoform in stem intestinal cells, whereas IR-B was predominant in differentiated enterocytes. Moreover, the IR-B expression was reduced in precancerous adenomas compared to normal colon tissue, as well as in aggressive, poorly differentiated human colorectal cancer cell lines compared to differentiated colorectal cancer cells, suggesting that IR-B could limit intestinal cell proliferation and transformation [44]. In contrast, in subjects with colorectal adenoma or in adenoma-free controls no difference was found in total IR mRNA and in the IR-A:IR-B ratio. However, using a logistic regression model, subjects having both high insulin level and increased IR-A:IR-B ratio were more likely to have adenomas than individuals with low plasma insulin [61].

Finally, in colorectal carcinoma specimens having an increased IR-A:IR-B ratio the expression of both IR isoforms was higher than that of the epidermal growth factor receptor (EGFR), a clinically validated target in this tumor type [62].

An increased IR-A expression and IGF-2 production was observed in primary cultures of thyroid cancer cells and in thyroid cancer specimens compared to normal thyroid cells (Table 1) [46]. The IR phosphorylation, due to increased IGF-2 activates a growth-promoting autocrine loop in cancer cells. This mechanism, together with alterations of the p53 family proteins, becomes predominant in poorly differentiated and anaplastic thyroid carcinomas compared to well-differentiated carcinomas [46,63].

The abnormal increase of both IR-A and IGF-1R was also found in progenitor/stem-like cells cultured as thyrospheres, and markedly decreased during differentiation when IR-B became predominant [47].

The IR isoforms play a role also in bone pathophysiology. Human mature osteoblasts mainly express IR-B whereas osteoblast precursors predominantly express IR-A, with a IR-B/IR-A mRNA ratio increasing along the osteogenic differentiation [64]. When bone precursor cells were transfected to overexpress IR-A, an increased proliferation rate was observed, while conversely, IR-B overexpression reduced cell growth. Therefore, IR-B isoforms seem to be involved in osteoblast precursor differentiation [64]. A very high level of IR-A expression was observed in both tissue specimens and cell lines of human osteosarcoma. Since osteosarcoma cells produce high levels of IGF-2, the biological role of the autocrine loop IGF-2/IR-A may have a relevant role and should be considered in the treatment strategy of osteosarcoma [48]. IR-A is the predominant isoform also in other types of sarcoma as leiomyosarcoma and rhabdomyosarcoma [65,66]. In a leiomyosarcoma cell line, without the interfering activity of IGF-1R, IGF-2 mediates cell growth and cell invasion through the predominantly expressed IR-A [66]. A similar role of the IR-A/IGF-2 pathway was detected also in solitary fibrous tumors, mesenchymal tumors frequently associated with hypoglycemia [67].

The IR-A isoform overexpression may have a significant detrimental role in all hyperinsulinemic conditions. The cancer-promoting effect of hyperinsulinemia is based on the knowledge that insulin has both metabolic and mitogenic actions via its own receptor in addition to the IGF-1R, which is activated by insulin with a much lower affinity. In cancer cells, the biological response to insulin cannot be predicted only on the basis of the IR and IGF-1R expression levels. In vitro studies indicate that in different cancer cell lines with different expression of the insulin and insulin-like receptors, insulin stimulates proliferation in all cell lines, but with different efficacy not necessarily related to the receptor content. This is explained by the presence of other factors (genetic and environmental) that also affect cell growth [68]. Insulin resistance is known to depend mainly by malfunctioning cell metabolic pathways [69]. The compensatory hyperinsulinemia to overcame the metabolic pathway resistance over-stimulates the mitogenic pathway and promotes cell proliferation. This deleterious mitogenic effect of hyperinsulinemia is more marked in cancer cells because malignant cells, as already documented, often overexpress the IR. Within the increased IR, in cancer the IR-A isoform is predominant, potentiating the cell proliferation, because of having a more marked mitogenic effect than IR-B, and also because having a high-affinity for IGF-2. Insulin resistance and hyperinsulinemia, therefore, are the main pathogenetic mechanisms why conditions like obesity and diabetes are at increased risk of cancer [70]. 

## 3. Mechanisms of Dysregulated IR Expression in Cancer

Given the complexity of the regulatory network involved in IR expression and IR isoforms generation, the aberrant expression of IR in cancer may occur at different levels. Studies of factors determining IR expression and either isoform predominance under different cellular conditions have focused on mRNA transcription and protein degradation as possible regulating mechanisms. In addition, IR isoform preferential expression has been mainly associated with alterations of the splicing factor machinery.

### 3.1. Alteration of Transcription

#### Transcription Factors

Regulation of *IR* gene expression is mainly attained at the transcriptional level. *IR* gene transcription is dependent on a number of stimulatory nuclear proteins, including Sp1 and HMGA1 [71,72,73]. In addition, *IR* gene transcription is functionally inactivated by its negative regulator p53. Indeed, p53 suppresses the IR promoter activity [74,75] and this finding may explain, at least in part, why IRs are also overexpressed in most human carcinomas having mutated p53 [76].

Interactions between stimulatory and inhibitory transcription factors play an important role in *IR* gene regulation and, therefore, may have a significant influence on the proliferative status of the cell. Alterations of IR transcription factors are very common in cancer cells and determine overall IR overexpression rather than either isoform preferential expression. Specifically, upregulation of Sp1 and HMGA1 or inactivation of p53 may be responsible for the increased IR cellular content. In addition, the IR mRNA up-regulation induced by serum deprivation was shown to involve the transcription factor FOXO1 (Table 2). [77]. Other mechanisms, frequently activated in cancer, can also contribute to IR overexpression in malignant cells.

### 3.2. Post-Transcriptional Dysregulation

#### 3.2.1. Alternative Splicing Regulatory Factors

IR is regulated at post-transcriptional levels by RNA-binding proteins (RBPs). These factors mainly regulate pre-mRNA alternative splicing. Upon transcription, pre-mRNA is processed, exons are joined, and a complex of ribonucleoproteins called spliceosome to remove introns [86,87]. Most human genes containing more than one exon undergo alternative splicing to generate multiple mature mRNA variants from a single pre-mRNA [88,89]. Through alternative, splicing identical pre-mRNA molecules are spliced in different forms. This mechanism is important in normal development for creating protein diversity [90] and providing mechanisms for the regulation of cell response to external and internal stimuli. Alterations of splicing programs appear to be crucially linked to the biological specificity of cancer cells.

**Figure 2:**
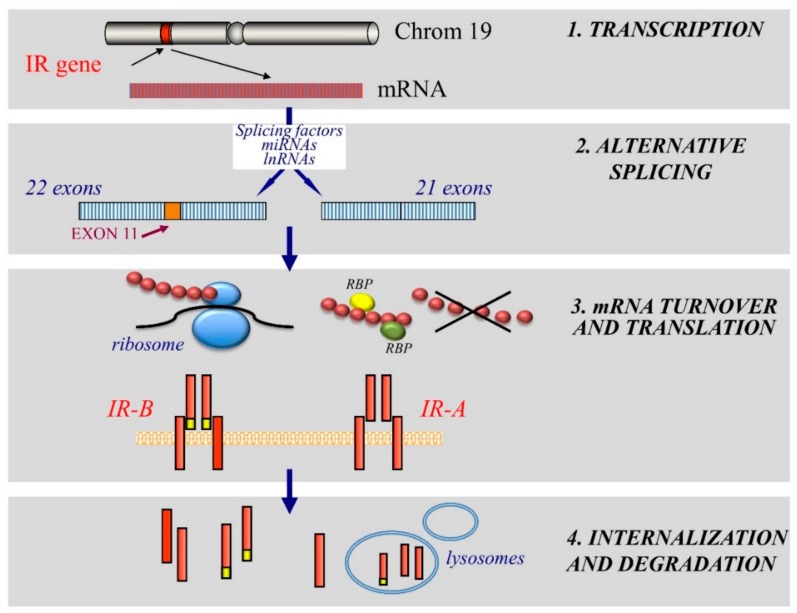
Schematic representation of mechanisms regulating the expression of IR isoforms. The figure summarizes the principal phases determining the level of the total cellular IR content and the relative IR isoform expression. (1) *Transcription regulation:* The balance between stimulatory and inhibitory transcription factors plays an important role in regulating *IR* gene transcription in chromosome 19; (2) *Alternative splicing:* At post-transcriptional level alternative splicing of the IR mRNA transcript (including or excluding exon 11) regulates the IR isoform relative abundance. Different modulators, such as splicing factors, miRNAs and lnRNAs are involved; (3) *mRNA turnover and translation:* The stability of IR mRNAs and its translation into the IR protein by ribosomes is regulated by the RNA binding proteins (RBPs); (4) *Internalization and degradation:* IR isoforms undergo internalization and protein degradation by ubiquitination at a different rate, through a process that facilitates endocytosis and endosomal degradation. The ligand binding feature is an important determinant of this process.

As splicing enhancer and silencer elements are responsible for the alternatively spliced *IR* intron 10 and exon 11 [91], several splicing factors have been hypothesized to have regulatory roles in the exon inclusion/exclusion generating the A and B isoforms of the IR protein (Figure 2) [92]. These findings are based on the correlation between the alternative splicing of IR mRNA and the expression of these splicing factors. IR isoform prevalence is associated with altered expression of mRNAs encoding for IR splicing enhancers and silencer in many cancer cell models [44].

Proteins involved in IR splicing includes CUG-BP1, hnRNPs, SR (serin/arginine-rich) proteins, and MBNL) proteins (Table 2).

(a) CUG-BP1 is a splicing silencer that facilitates exon 11 exclusion, favoring IR-A expression [91]. It plays also a role in the regulation of editing and translation [93,94].
(b) hnRNP family proteins are involved in pre-mRNA splicing, mRNA export, stability and translation [95,96]. hRNP H inhibits IR exon 11 splicing [79,97] and interacts with CUG-BP1 promoting a maximum inhibition of IR exon 11 inclusion. hnRNP A1 inhibits, while hnRNP F enhances, inclusion of exon 11 [80]. It has been shown that hnRNPA2/B1 is overexpressed in human hepatocellular carcinoma (HCC) tissues, but not in normal liver tissues. Moreover, when cancer dedifferentiates, nuclear hnRNPA2/B1 translocates from the nucleus to the cytoplasm [81].
(c) SR (serin/arginine-rich) proteins are alternative splicing regulatory proteins that promote exon inclusion. They also regulate mRNA export and translation [98]. SRSF1 belongs to this family and compete with hnRNP A1 for the same splicing site. Together with SRp20 it promotes exon inclusion recognizing exon splicing enhancers in exon 11 [91]. Loss of SRp20/SRSF3 has been associated with increased IGF-2 and IR-A, which promotes proliferation, Wnt/b-catenin activation and induction of c-Myc along with the promotion of aberrant splicing and epithelial mesenchymal transition (EMT) genes’ expression. In a significant proportion of cancers SRp20/SRSF3 is absent or mutated, suggesting that this mechanism may contribute to the high IR-A:IR-B ratio in cancer cells (Table 2) [82]. Interestingly, insulin stimulation may induce proteasome-dependent degradation of SRp20/SRSF3, which in turn may favor IGF-2 and IR-A increase.
(d) Muscleblind-like (MBNL) proteins are splicing enhancers involved in the alternative splicing of pre-mRNAs [99]. Antagonizing the action of CUG-BP1 [79,100], and interacting with other splicing regulators (such as hnRNP H), MBNL1 promotes exon 11 inclusion and favors IR-B isoform [101,102]. Indeed, downregulation of MBNL1 and upregulation of CUG-BP1 are associated with reduced IR-B levels [79,103], demonstrating a crucial role for these RNA-binding proteins in both IR-B expression and insulin sensitivity. Different splicing enhancers, such as MBNL1, MBNL2 and SRSF3, promote exon 11 inclusion favoring IR-B expression, while CUG-BP1 is a silencer that promotes exon 11 exclusion thus supporting IR-A predominance (Table 2) [91,102].

In this complex network, different mutations of splicing factors have been found in different cancers, not always correlated with changes in the relative abundance of IR isoforms. In addition, no specific mutation of splicing factors has been found to correlate with increased IR-A:IR-B ratio.

#### 3.2.2. Non-Coding RNAs

Non-coding RNAs, including miRNAs and long non-coding RNAs (lncRNAs), play an important role in post-transcriptional regulation of gene expression [104,105].

miRNAs are small non-coding RNAs able to modulate gene expression at the post-transcriptional level. They act through destabilization of mRNA transcripts and/or repression of translation mainly by base-pairing to the 3′-untranslated regions (UTRs) of the target mRNAs, therefore either triggering degradation of the target mRNAs or suppressing its translation [106]. A growing body of evidence has suggested that miRNAs are involved in many cellular processes, such as cell proliferation, apoptosis and metabolism [107]. They may also behave as tumor suppressors and, consequently, the dysregulation of miRNAs expression can promote human diseases, including cancer [108]. Mechanisms accounting for the low miRNA expression in various tumors include DNA copy number reduction and hypermethylation of the CpG islands upstream of the miRNA gene [109]. Different miRNAs have been reported to be involved in IR modulation and their alteration in cancer has been associated with IR up-regulation and with IR-A:IR-B ratio increase.

miRNAs involved in IR regulation, such as miR-195, Let-7, miR-103/107 and miR-424 act as tumor suppressors with anti-proliferative and pro-differentiation effects [14]. As a consequence, their expression levels are often down-regulated in cancer cells and associated with cancer progression, EMT and stem features of malignant cells, as well as with multidrug resistance.

Various miRNAs are also involved in the regulation of the main protein cascades of the insulin signaling pathway, thus affecting insulin resistance beyond the development and progression of cancer. However, insulin resistance, characterized by hyperinsulinemia, is considered a determinant of a potential cancer initiation factor and a determinant element for cancer progression [110].

Recently, miRNAs have emerged as inhibitors of glucose metabolism, mitochondrial respiration and cell proliferation by targeting IR and IRS-1 inhibition, as in the case of mir-128 in triple-negative breast cancer cell models [83]. Noteworthy, miRNAs have multiple rather than single targets and, therefore, several effects can occur from a single alteration. For instance, miR-15b/16-2 modulates not only IR, but also the IGF-1R and cyclin D genes: Its down-regulation has been associated with a B-cell malignancy with the characteristics of the human chronic lymphocytic leukemia, probably as a result of multiple effects (Table 2) [84].

Finally, dysregulation of microRNAs may influence splicing factors. Several lines of evidence have demonstrated an aberrant expression of splicing factors in cancer cells [111]. Splicing factors are often a direct target of miRNAs [111] and, conversely, miRNAs may also affect splicing factors. Furthermore, SR proteins can be regulated at transcriptional level by miRNAs. For instance, the tumor suppressor miR-1 induces apoptosis of bladder cancer cells inhibiting the splicing factor serine/arginine-rich 9 (SRSF9/SRp30c) [85].

The miRNAs dysregulation that is often observed in cancer cells, therefore, is involved in the altered IR expression and the abnormal IR isoform ratio typical of most cancer cells (Table 2).

At variance with miRNA, lncRNAs regulate gene expression via epigenetic mechanisms. They are involved in the splicing processes, mRNA stability and translation by binding to miRNAs or RBPs [112]. Although lncRNAs are not directly involved in post-transcriptional regulation of the insulin system, some of them have been associated with β-cell development [113]. In addition, the lncRNA CRNDE (colorectal neoplasia differentially expressed) has been recently described to be repressed by insulin/IGFs treatment [114]. CRNDE seems to be a downstream target of PI3K/AKT and MAPK pathways capable to influence glucose and lipid metabolisms. Indeed, its expression promotes the metabolic changes by which cancer cells switch to aerobic glycolysis [114]. However, future studies are needed to better understand their roles in cancer.

### 3.3. Regulation of mRNA Turnover and Translation by RNA Binding Proteins (RBPs)

The regulation of mRNA translation into the IR protein by ribosome is also an important mechanism determining the level of cell IR content. This process is regulated by proteins that control mRNA translation and stability. Usually, translation is initiated in a cap-dependent manner, since cap recognition at the 5′ end of RNA molecules is required for the assembly of the initiation complex. However, there is also a cap-independent initiation of translation that involves an IRES (internal ribosome entry segment) sequence located in the 5′UTR of the RNA. The mRNA encoding human IR contains functional IRES, which is dependent on PTB (polypiryimidine tract binding) protein for the activity in vitro e in vivo [78,115,116]. Cells may use IR-IRES to increase translation under conditions where cap-dependent initiation is compromised (Table 2). The activity of IR-IRES differs in different cells and is stimulated by insulin [78]. Moreover, translation initiation of a number of mRNAs is specifically regulated during differentiation, growth and stress [117]. Although the proteins required for this process have yet to be fully identified, only PTBPs have shown to regulate IR mRNA translation by binding to the 5’UTR and enhancing IRES-mediated IR mRNA translocation to the ribosomes (Figure 2) [78].

### 3.4. IR Degradation

Previous studies on insulin-resistance have shown that dysregulation of post-receptor insulin signaling can be influenced by decreased IR tyrosine kinase activity and decreased cell membrane IR expression. Likewise, IR expression on the cell membrane could be determined by the internalization rate and enhanced protein degradation (Figure 2) [118]. Several E3 ubiquitin ligases regulate IR and IRS protein expression by ubiquitination, facilitating endocytosis and endosomal degradation.

Recently, MARCH1, an E3 ubiquitin ligase and a new repressor of IR signaling, has been found to control IR membrane stability by polyubiquitination through the transcription factor FOXO1 [119]. Consistent with this hypothesis MARCH1 down-regulation is associated with increased IR plasma membrane content. In cancer cells having an activated IR-A/IGF-2 loop, reduced MARCH1 levels could be responsible of the increased IR expression levels on the cells surface, thus potentiating the IR-mediated mitogenic effects.

## 4. Additional Roles of IR Alterations in Cancer Biology

### 4.1. Involvement in Cancer Cell Stemness

Recently, IR and it’s signaling pathway have been demonstrated to play a critical role in the regulation of cancer stem cell functions [23,47]. Activated IR-A/IGF-2 loop may support the stem cell ability of self-renewal, promoting cancer cell survival and migration [47]. Interestingly, in immortalized and in malignant cells, IR activation regulates several transcription factors involved in the EMT process and in pluripotency, such as p53, Oct-4, and Nanog. Indeed, the IR axis induces the phosphorylation and inactivation of p53, a negative regulator of Oct-4 and Nanog [120] thus removing p53 suppression of stem cell typical markers.

Several studies indicated that the IGF-2/IR-A axis has a predominant role in some tumors. The activated IGF-2/IR-A loop is associated with de-differentiation and stem-like phenotype and also in EMT [121] and other stem-like features [47], which play a key role in cancer development and recurrence. In human thyroid cancer, cells cultured as thyrospheres and acquiring stem-like features, have an up-regulation of the IGF-2/IR-A loop, strongly associated with tumor aggressiveness and loss of differentiation [46]. When comparing thyrospheres obtained from human thyroid specimens of either cancer or normal thyroid tissue, the stem-like phenotype and self-renewal ability was significantly increased in progenitor/stem malignant cells when compared with normal thyrospheres or differentiated thyrocytes [47,122]. In stem/progenitor thyroid cancer cells, the IGF-2/IR-A loop was up-regulated [47]. Recently, DDR1 was found to be an important regulator of both IGF-1R and IR function and expression [123,124,125,126]. So far, as IR-A is the prevalent IR isoform expressed in the most aggressive tumors, DDR-1 can behave as a potential modulator of the IGF-2/IR-A loop [127]. 

### 4.2. Correlation with Advanced Cancer Disease

It is today recognized that the IR is overexpressed in many tumors [17,33,38,46,66] and that also IGF-2 and IR-A overexpression is present in a wide range of human cancers and is associated with a poor prognosis [128,129,130]. In addition, IR-A activation, mainly by IGF-2, promotes metastasis [121] and is linked to tumor progression and de-differentiation. Indeed, IR expression is very high in poorly differentiated and anaplastic cancers [46]. The presence of both high levels of IR-A and hyperinsulinemia correlates with more aggressive and hormone-resistant breast cancers. As already mentioned, IR-A activation in cancer cells is associated with active downstream signaling pathway with predominant mitogenic effect [14] and poor patient prognosis. Many studies have demonstrated the role of insulin-resistance associated with metabolic disorders, such as obesity and type 2 diabetes mellitus (T2DM) in carcinogenesis and cancer progression [14,27,131,132]. In conclusion, the overexpressed IR and its predominantly mitogenic isoform can produce, in all conditions of hyperinsulinemia, a series of deleterious effects, including EMT stimulation, cell migration, angiogenesis, and resistance to chemotherapeutic agents.

### 4.3. The IR Implication in Resistance to Targeted Therapies

Intrinsic and acquired resistance to targeted therapies frequently results from the activation of alternative tyrosine kinase receptors (RTKs). The IR, for instance, may be implicated in the resistance to anti-IGF-1R therapies used as both in monotherapy, or in combination with cytotoxic agents. Indeed, many evidences indicate that a compensatory crosstalk between IGF-1R and the IR may account for resistance to IGF-1R targeted therapy [133,134], since genetic, shRNA-mediated or pharmacological inactivation of the IGF-1R can result in IR up-regulation [133,135,136,137]. IR, therefore, can mediate primary resistance to IGF-1R targeted therapy and can be used as a potential biomarker for patient selection. These findings provide a rationale for co-targeting IGF-1R and IR in cancer treatment. At this regard dual inhibitors, such as OSI-906, have already been developed and some of them have already been taken into clinical trials [138]. However, drugs targeting the IGF axis have shown different limitations as they may induce insulin resistance and a compensatory hyperinsulinemia, negatively affecting cancer progression. At this regard, new therapeutic approaches inhibiting cell growth and proliferation by affecting the IGF axis have been proposed [139,140]. A recent study has suggested an alternative approach, targeting both IGF-1R and IR degradation to treat cancers having IR/IGF-1R overactivation [141]. By an artificial E3 ubiquitin ligase able to recognize proteins of interest, a method to increase ubiquitination-mediated proteolysis has been proposed using an engineered ubiquitin ligase composed of an IGF-1R/IR binding domain (the PTB domain of IRS-1) and a functional ubiquitin ligase domain. Such a strategy may have many advantages as most types of cancer cells overexpress more than one oncoprotein, a mechanism able to compensate and favor survival pathways to overcome single-target directed therapy. This degradation strategy could be combined with other inhibitors and chemotherapeutic agents.

## 5. Conclusions

Both IR isoforms are overexpressed in many cancer types and the IR-A:IR-B ratio in most cases is in favor of the IR-A isoform. The prevalence of IR-A contributes to modifying the response of cancer cells to insulin and IGFs by different mechanisms.

First, IR overexpression sensitizes cancer cells to the pleiotropic effects of insulin, in particular in all clinical conditions with hyperinsulinemia.

Second, IR-A binds to IGF-2 with high affinity. Both cancer and stromal cells produce IGF-2 and therefore IR-A, in addition to IGF-1R, mediates the mitogenic and antiapoptotic effects of IGF-2.

Third, IR-A may form hybrid receptors with IGF-1R, causing subtle, but biologically significant differences in ligand-stimulated signaling. A high IR-A:IGF-1R ratio may favor the effects of IGF-2 over the effects of IGF-1. After the failures of anti-IGF-1R trials in some cancer types, IR-A signaling has been postulated as one of the major mediators of resistance to anti-IGF-1R therapy. Moreover, IGF-2 production may be stimulated by antitumor treatments and may enhance drug resistance through IR-A binding.

The mechanisms of IR isoform dysregulation in cancer are complex and only partially understood. Studies have focused on mRNA transcription, splicing factors and receptor protein degradation. Alterations of one or more of these mechanisms seem to be involved in the IR isoform overexpression. 

The role of the IR isoforms in the clinical setting of cancer biology is not yet fully defined and deserves further studies. One difficult for IR isoform study is dependent from the unavailability of antibodies able to distinguish the two IR isoforms. Consequently, the expression of the IR isoforms in cancer cannot be detected at the protein level. However, in order to promote a better understanding of cancer biology, the role of IR isoforms in malignant cells must be evaluated as a relevant factor in a complex system where other factors are aberrantly activated.

In conclusion, novel anti-cancer therapeutic strategies should be established for a new therapeutic approach, including the inhibition of the IR-A overexpression, the increased IR-A:IR-B and the IR-A/IGF-1R expression in cancer cells.

## Figures and Tables

**Table 1:** Insulin receptor and insulin receptor isoforms expression in various cancer types in comparison with non-malignant paired tissues.

Cancer Type	Model	IR	IR-A	IR-B	IR-A:IR-B Ratio	References
**Hormone-dependent**						
Breast	h-BC specimens	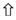				[28]
	h-BC cell lines and specimens		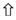			[17,29]
	h-BC specimens		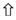	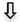	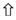	[30]
	h-BC specimens		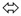	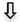	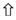	[31]
Prostate	h-PC specimens	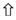				[32,33]
	h-PC specimens				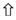	[34]
	h-PC cell lines		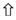			[35]
Endometrial	h-EC cell lines and specimens	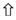	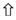			[36]
	h-EC specimens		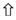	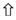		[37]
Ovarian	h-OV cell lines	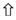	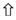			[38]
**Hormone- independent**						
Liver	h-HCC specimens	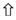	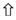	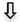	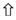	[39]
	r-HCC specimens	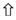				[40]
	m-HCC specimens	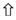	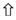			[41]
Bladder	h-BLC specimens	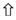				[42]
Lung	h-NSCLC specimens			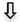	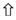	[43]
	h-LC specimens		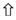			[17]
Colon	m-PCA, h-CC cell lines			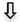		[44]
	h-CC specimens		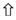			[17]
Thyroid	h-TC specimens	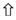				[45]
	h-TC cell lines and specimens	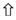	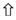			[46]
	h-TC cell lines		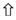			[47]
Osteosarcoma	h-OS cell lines and specimens		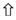			[48]

h, human; r, rat; m, mice; BC, breast cancer; HCC, hepatocarcinoma; PC, prostate cancer; BLC, bladder cancer; EC, endometrial cancer; OC, ovarian cancer; NSCLC, non-small cell lung cancer; LC, lung cancer; CC, colon cancer; PCA, precancerous colon adenoma; TC, thyroid cancer; OS, osteosarcoma.

**Table 2:** Molecular mechanisms associated with IR dysregulated expression.

Mechanism of Altered IR Expression	Dysregulation	References
IR transcription factors upregulation (Sp1, HMGA1, or FOXO1)	IR upregulation	[73]
p53 inactivation	IR upregulation	[75]
Enhanced IRES-mediated IR mRNA translocation to the ribosomes	IR upregulation	[78]
CUG-BP1 increase	Increased IRA:IRB ratio	[79]
hRNP H increase	Increased IRA:IRB ratio	[79]
hRNP A1 increase	Increased IRA:IRB ratio	[80]
hRNP A2/B1 increase	Increased IRA:IRB ratio	[81]
Loss of SRSF3 and SRp20	Increased IGF2 and IRA:IRB ratio	[82]
MBNL downregulation	Increased IRA:IRB ratio	[79]
mir-128 downregulation	Increased IRA:IRB ratio	[83]
mir-15b/16-2 downregulation	Increased IRA:IRB ratio	[84]
mir-1 downregulation	Increased IRA:IRB ratio	[85]

## References

[1] Tatulian S.A. (2015). Structural dynamics of insulin receptor and transmembrane signaling. Biochemistry.

[2] Frasca F., Pandini G., Sciacca L., Pezzino V., Squatrito S., Belfiore A., Vigneri R. (2008). The role of insulin receptors and IGF-I receptors in cancer and other diseases. Arch. Physiol. Biochem..

[3] Lawrence M.C., McKern N.M., Ward C.W. (2007). Insulin receptor structure and its implications for the IGF-I receptor. Curr. Opin. Struct. Biol..

[4] De Meyts P. (2008). The insulin receptor: A prototype for dimeric, allosteric membrane receptors?. Trends Biochem. Sci..

[5] Taniguchi C.M., Emanuelli B., Kahn C.R. (2006). Critical nodes in signalling pathways: Insights into insulin action. Nat. Rev. Mol. Cell Biol..

[6] Zaid H., Antonescu C.N., Randhawa V.K., Klip A. (2008). Insulin action on glucose transporters through molecular switches, tracks and tethers. Biochem. J..

[7] McKern N.M., Lawrence M.C., Streltsov V.A., Lou M.Z., Adams T.E., Lovrecz G.O., Elleman T.C., Richards K.M., Bentley J.D., Pilling P.A. (2006). Structure of the insulin receptor ectodomain reveals a folded-over conformation. Nature.

[8] Sciacca L., Prisco M., Wu A., Belfiore A., Vigneri R., Baserga R. (2003). Signaling differences from the A and B isoforms of the insulin receptor (IR) in 32D cells in the presence or absence of IR substrate-1. Endocrinology.

[9] Kaplan S.A. (1984). The insulin receptor. J. Pediatr..

[10] Hernandez-Sanchez C., Mansilla A., de Pablo F., Zardoya R. (2008). Evolution of the insulin receptor family and receptor isoform expression in vertebrates. Mol. Biol. Evol..

[11] Whittaker J., Groth A.V., Mynarcik D.C., Pluzek L., Gadsboll V.L., Whittaker L.J. (2001). Alanine scanning mutagenesis of a type 1 insulin-like growth factor receptor ligand binding site. J. Biol. Chem..

[12] Ullrich A., Gray A., Tam A.W., Yang-Feng T., Tsubokawa M., Collins C., Henzel W., Le Bon T., Kathuria S., Chen E. (1986). Insulin-like growth factor 1 receptor primary structure: Comparison with insulin receptor suggests structural determinants that define functional specificity. EMBO J..

[13] Belfiore A., Frasca F., Pandini G., Sciacca L., Vigneri R. (2009). Insulin receptor isoforms and insulin receptor/insulin-like growth factor receptor hybrids in physiology and disease. Endocr. Rev..

[14] Belfiore A., Malaguarnera R., Vella V., Lawrence M.C., Sciacca L., Frasca F., Morrione A., Vigneri R. (2017). Insulin receptor isoforms in physiology and disease: An updated view. Endocr. Rev..

[15] Westermeier F., Saez T., Arroyo P., Toledo F., Gutierrez J., Sanhueza C., Pardo F., Leiva A., Sobrevia L. (2016). Insulin receptor isoforms: An integrated view focused on gestational diabetes mellitus. Diabetes Metab. Res. Rev..

[16] Denley A., Wallace J.C., Cosgrove L.J., Forbes B.E. (2003). The insulin receptor isoform exon 11-(IR-A) in cancer and other diseases: A review. Horm. Metab. Res..

[17] Frasca F., Pandini G., Scalia P., Sciacca L., Mineo R., Costantino A., Goldfine I.D., Belfiore A., Vigneri R. (1999). Insulin receptor isoform A, a newly recognized, high-affinity insulin-like growth factor II receptor in fetal and cancer cells. Mol. Cell. Biol..

[18] Morcavallo A., Gaspari M., Pandini G., Palummo A., Cuda G., Larsen M.R., Vigneri R., Belfiore A. (2011). Research resource: New and diverse substrates for the insulin receptor isoform a revealed by quantitative proteomics after stimulation with IGF-II or insulin. Mol. Endocrinol..

[19] Sciacca L., Cassarino M.F., Genua M., Pandini G., Le Moli R., Squatrito S., Vigneri R. (2010). Insulin analogues differently activate insulin receptor isoforms and post-receptor signalling. Diabetologia.

[20] Yamaguchi Y., Flier J.S., Benecke H., Ransil B.J., Moller D.E. (1993). Ligand-binding properties of the two isoforms of the human insulin receptor. Endocrinology.

[21] Benyoucef S., Surinya K.H., Hadaschik D., Siddle K. (2007). Characterization of insulin/IGF hybrid receptors: Contributions of the insulin receptor L2 and Fn1 domains and the alternatively spliced exon 11 sequence to ligand binding and receptor activation. Biochem. J..

[22] Blanquart C., Achi J., Issad T. (2008). Characterization of IRA/IRB hybrid insulin receptors using bioluminescence resonance energy transfer. Biochem. Pharmacol..

[23] Malaguarnera R., Belfiore A. (2014). The emerging role of insulin and insulin-like growth factor signaling in cancer stem cells. Front. Endocrinol. (Lausanne).

[24] Pandini G., Medico E., Conte E., Sciacca L., Vigneri R., Belfiore A. (2003). Differential gene expression induced by insulin and insulin-like growth factor-II through the insulin receptor isoform A. J. Biol. Chem..

[25] Janssen J.A., Varewijck A.J. (2014). IGF-IR targeted therapy: Past, present and future. Front. Endocrinol. (Lausanne).

[26] Belfiore A., Malaguarnera R. (2011). Insulin receptor and cancer. Endocr. Relat. Cancer.

[27] Vigneri R., Goldfine I.D., Frittitta L. (2016). Insulin, insulin receptors, and cancer. J. Endocrinol. Investig..

[28] Papa V., Pezzino V., Costantino A., Belfiore A., Giuffrida D., Frittitta L., Vannelli G.B., Brand R., Goldfine I.D., Vigneri R. (1990). Elevated insulin receptor content in human breast cancer. J. Clin. Investig..

[29] Sciacca L., Costantino A., Pandini G., Mineo R., Frasca F., Scalia P., Sbraccia P., Goldfine I.D., Vigneri R., Belfiore A. (1999). Insulin receptor activation by IGF-II in breast cancers: Evidence for a new autocrine/paracrine mechanism. Oncogene.

[30] Aljada A., Saleh A.M., Al-Aqeel S.M., Shamsa H.B., Al-Bawab A., Al Dubayee M., Ahmed A.A. (2015). Quantification of insulin receptor mRNA splice variants as a diagnostic tumor marker in breast cancer. Cancer Biomark..

[31] Huang J., Morehouse C., Streicher K., Higgs B.W., Gao J., Czapiga M., Boutrin A., Zhu W., Brohawn P., Chang Y. (2011). Altered expression of insulin receptor isoforms in breast cancer. PLoS ONE.

[32] Breen K.J., O’Neill A., Murphy L., Fan Y., Boyce S., Fitzgerald N., Dorris E., Brady L., Finn S.P., Hayes B.D. (2017). Investigating the role of the IGF axis as a predictor of biochemical recurrence in prostate cancer patients post-surgery. Prostate.

[33] Cox M.E., Gleave M.E., Zakikhani M., Bell R.H., Piura E., Vickers E., Cunningham M., Larsson O., Fazli L., Pollak M. (2009). Insulin receptor expression by human prostate cancers. Prostate.

[34] Heni M., Hennenlotter J., Scharpf M., Lutz S.Z., Schwentner C., Todenhofer T., Schilling D., Kuhs U., Gerber V., Machicao F. (2012). Insulin receptor isoforms A and B as well as insulin receptor substrates-1 and -2 are differentially expressed in prostate cancer. PLoS ONE.

[35] Perks C.M., Zielinska H.A., Wang J., Jarrett C., Frankow A., Ladomery M.R., Bahl A., Rhodes A., Oxley J., Holly J.M. (2016). Insulin receptor isoform variations in prostate cancer cells. Front. Endocrinol. (Lausanne).

[36] Wang C.F., Zhang G., Zhao L.J., Qi W.J., Li X.P., Wang J.L., Wei L.H. (2013). Overexpression of the insulin receptor isoform A promotes endometrial carcinoma cell growth. PLoS ONE.

[37] Flannery C.A., Saleh F.L., Choe G.H., Selen D.J., Kodaman P.H., Kliman H.J., Wood T.L., Taylor H.S. (2016). Differential expression of IR-A, IR-B and IGF-1R in endometrial physiology and distinct signature in adenocarcinoma. J. Clin. Endocrinol. Metab..

[38] Kalli K.R., Falowo O.I., Bale L.K., Zschunke M.A., Roche P.C., Conover C.A. (2002). Functional insulin receptors on human epithelial ovarian carcinoma cells: Implications for IGF-II mitogenic signaling. Endocrinology.

[39] Chettouh H., Fartoux L., Aoudjehane L., Wendum D., Claperon A., Chretien Y., Rey C., Scatton O., Soubrane O., Conti F. (2013). Mitogenic insulin receptor-A is overexpressed in human hepatocellular carcinoma due to EGFR-mediated dysregulation of RNA splicing factors. Cancer Res..

[40] Aleem E., Nehrbass D., Klimek F., Mayer D., Bannasch P. (2011). Upregulation of the insulin receptor and type I insulin-like growth factor receptor are early events in hepatocarcinogenesis. Toxicol. Pathol..

[41] Sakurai Y., Kubota N., Takamoto I., Obata A., Iwamoto M., Hayashi T., Aihara M., Kubota T., Nishihara H., Kadowaki T. (2017). Role of insulin receptor substrates in the progression of hepatocellular carcinoma. Sci. Rep..

[42] Roudnicky F., Dieterich L.C., Poyet C., Buser L., Wild P., Tang D., Camenzind P., Ho C.H., Otto V.I., Detmar M. (2017). High expression of insulin receptor on tumour-associated blood vessels in invasive bladder cancer predicts poor overall and progression-free survival. J. Pathol..

[43] Jiang L., Zhu W., Streicher K., Morehouse C., Brohawn P., Ge X., Dong Z., Yin X., Zhu G., Gu Y. (2014). Increased IR-A/IR-B ratio in non-small cell lung cancers associates with lower epithelial-mesenchymal transition signature and longer survival in squamous cell lung carcinoma. BMC Cancer.

[44] Andres S.F., Simmons J.G., Mah A.T., Santoro M.A., Van Landeghem L., Lund P.K. (2013). Insulin receptor isoform switching in intestinal stem cells, progenitors, differentiated lineages and tumors: Evidence that IR-B limits proliferation. J. Cell Sci..

[45] Frittitta L., Sciacca L., Catalfamo R., Ippolito A., Gangemi P., Pezzino V., Filetti S., Vigneri R. (1999). Functional insulin receptors are overexpressed in thyroid tumors: Is this an early event in thyroid tumorigenesis?. Cancer.

[46] Vella V., Pandini G., Sciacca L., Mineo R., Vigneri R., Pezzino V., Belfiore A. (2002). A novel autocrine loop involving IGF-II and the insulin receptor isoform-A stimulates growth of thyroid cancer. J. Clin. Endocrinol. Metab..

[47] Malaguarnera R., Frasca F., Garozzo A., Giani F., Pandini G., Vella V., Vigneri R., Belfiore A. (2011). Insulin receptor isoforms and insulin-like growth factor receptor in human follicular cell precursors from papillary thyroid cancer and normal thyroid. J. Clin. Endocrinol. Metab..

[48] Avnet S., Sciacca L., Salerno M., Gancitano G., Cassarino M.F., Longhi A., Zakikhani M., Carboni J.M., Gottardis M., Giunti A. (2009). Insulin receptor isoform A and insulin-like growth factor II as additional treatment targets in human osteosarcoma. Cancer Res..

[49] Mathieu M.C., Clark G.M., Allred D.C., Goldfine I.D., Vigneri R. (1997). Insulin receptor expression and clinical outcome in node-negative breast cancer. Proc. Assoc. Am. Physicians.

[50] Rostoker R., Abelson S., Bitton-Worms K., Genkin I., Ben-Shmuel S., Dakwar M., Orr Z.S., Caspi A., Tzukerman M., LeRoith D. (2015). Highly specific role of the insulin receptor in breast cancer progression. Endocr. Relat. Cancer.

[51] Wang C., Wang X., Gong G., Ben Q., Qiu W., Chen Y., Li G., Wang L. (2012). Increased risk of hepatocellular carcinoma in patients with diabetes mellitus: A systematic review and meta-analysis of cohort studies. Int. J. Cancer.

[52] Xu H., Lee M.S., Tsai P.Y., Adler A.S., Curry N.L., Challa S., Freinkman E., Hitchcock D.S., Copps K.D., White M.F. (2018). Ablation of insulin receptor substrates 1 and 2 suppresses Kras-driven lung tumorigenesis. Proc. Natl. Acad. Sci. USA.

[53] Frisch C.M., Zimmermann K., Zillessen P., Pfeifer A., Racke K., Mayer P. (2015). Non-small cell lung cancer cell survival crucially depends on functional insulin receptors. Endocr. Relat. Cancer.

[54] Vella V., Sciacca L., Pandini G., Mineo R., Squatrito S., Vigneri R., Belfiore A. (2001). The IGF system in thyroid cancer: New concepts. Mol. Pathol..

[55] Arcaro A., Doepfner K.T., Boller D., Guerreiro A.S., Shalaby T., Jackson S.P., Schoenwaelder S.M., Delattre O., Grotzer M.A., Fischer B. (2007). Novel role for insulin as an autocrine growth factor for malignant brain tumour cells. Biochem. J..

[56] Harrington S.C., Weroha S.J., Reynolds C., Suman V.J., Lingle W.L., Haluska P. (2012). Quantifying insulin receptor isoform expression in FFPE breast tumors. Growth Horm. IGF Res..

[57] Bjorner S., Rosendahl A.H., Simonsson M., Markkula A., Jirstrom K., Borgquist S., Rose C., Ingvar C., Jernstrom H. (2017). Combined and individual tumor-specific expression of insulin-like growth factor-I receptor, insulin receptor and phospho-insulin-like growth factor-I receptor/insulin receptor in primary breast cancer: Implications for prognosis in different treatment groups. Oncotarget.

[58] Heidegger I., Kern J., Ofer P., Klocker H., Massoner P. (2014). Oncogenic functions of IGF1R and INSR in prostate cancer include enhanced tumor growth, cell migration and angiogenesis. Oncotarget.

[59] Keku T.O., Lund P.K., Galanko J., Simmons J.G., Woosley J.T., Sandler R.S. (2005). Insulin resistance, apoptosis, and colorectal adenoma risk. Cancer Epidemiol. Biomark. Prev..

[60] Vidal A.C., Lund P.K., Hoyo C., Galanko J., Burcal L., Holston R., Massa B., Omofoye O., Sandler R.S., Keku T.O. (2012). Elevated C-peptide and insulin predict increased risk of colorectal adenomas in normal mucosa. BMC Cancer.

[61] Santoro M.A., Andres S.F., Galanko J.A., Sandler R.S., Keku T.O., Lund P.K. (2014). Reduced insulin-like growth factor I receptor and altered insulin receptor isoform mRNAs in normal mucosa predict colorectal adenoma risk. Cancer Epidemiol. Biomark. Prev..

[62] Forest A., Amatulli M., Ludwig D.L., Damoci C.B., Wang Y., Burns C.A., Donoho G.P., Zanella N., Fiebig H.H., Prewett M.C. (2015). Intrinsic resistance to cixutumumab is conferred by distinct isoforms of the insulin receptor. Mol. Cancer Res..

[63] Vella V., Puppin C., Damante G., Vigneri R., Sanfilippo M., Vigneri P., Tell G., Frasca F. (2009). DeltaNp73alpha inhibits PTEN expression in thyroid cancer cells. Int. J. Cancer.

[64] Avnet S., Perut F., Salerno M., Sciacca L., Baldini N. (2012). Insulin receptor isoforms are differently expressed during human osteoblastogenesis. Differentiation.

[65] Diaz L.E., Chuan Y.C., Lewitt M., Fernandez-Perez L., Carrasco-Rodriguez S., Sanchez-Gomez M., Flores-Morales A. (2007). IGF-II regulates metastatic properties of choriocarcinoma cells through the activation of the insulin receptor. Mol. Hum. Reprod..

[66] Sciacca L., Mineo R., Pandini G., Murabito A., Vigneri R., Belfiore A. (2002). In IGF-I receptor-deficient leiomyosarcoma cells autocrine IGF-II induces cell invasion and protection from apoptosis via the insulin receptor isoform A. Oncogene.

[67] Li Y., Chang Q., Rubin B.P., Fletcher C.D., Morgan T.W., Mentzer S.J., Sugarbaker D.J., Fletcher J.A., Xiao S. (2007). Insulin receptor activation in solitary fibrous tumours. J. Pathol..

[68] Sciacca L., Cassarino M.F., Genua M., Vigneri P., Giovanna Pennisi M., Malandrino P., Squatrito S., Pezzino V., Vigneri R. (2014). Biological effects of insulin and its analogs on cancer cells with different insulin family receptor expression. J. Cell. Physiol..

[69] Cusi K., Maezono K., Osman A., Pendergrass M., Patti M.E., Pratipanawatr T., DeFronzo R.A., Kahn C.R., Mandarino L.J. (2000). Insulin resistance differentially affects the PI 3-kinase- and MAP kinase-mediated signaling in human muscle. J. Clin. Investig..

[70] Sciacca L., Vigneri R., Tumminia A., Frasca F., Squatrito S., Frittitta L., Vigneri P. (2013). Clinical and molecular mechanisms favoring cancer initiation and progression in diabetic patients. Nutr. Metab. Cardiovasc. Dis..

[71] Aiello A., Pandini G., Sarfstein R., Werner H., Manfioletti G., Vigneri R., Belfiore A. (2010). HMGA1 protein is a positive regulator of the insulin-like growth factor-I receptor gene. Eur. J. Cancer.

[72] Chiefari E., Tanyolac S., Iiritano S., Sciacqua A., Capula C., Arcidiacono B., Nocera A., Possidente K., Baudi F., Ventura V. (2013). A polymorphism of HMGA1 is associated with increased risk of metabolic syndrome and related components. Sci. Rep..

[73] Foti D., Iuliano R., Chiefari E., Brunetti A. (2003). A nucleoprotein complex containing Sp1, C/EBP beta, and HMGI-Y controls human insulin receptor gene transcription. Mol. Cell. Biol..

[74] Ohlsson C., Kley N., Werner H., LeRoith D. (1998). P53 regulates insulin-like growth factor-I (IGF-I) receptor expression and IGF-I-induced tyrosine phosphorylation in an osteosarcoma cell line: Interaction between p53 and Sp1. Endocrinology.

[75] Webster N.J., Resnik J.L., Reichart D.B., Strauss B., Haas M., Seely B.L. (1996). Repression of the insulin receptor promoter by the tumor suppressor gene product p53: A possible mechanism for receptor overexpression in breast cancer. Cancer Res..

[76] Baserga R. (1994). Oncogenes and the strategy of growth factors. Cell.

[77] Puig O., Tjian R. (2005). Transcriptional feedback control of insulin receptor by dFOXO/FOXO1. Genes Dev..

[78] Spriggs K.A., Cobbold L.C., Ridley S.H., Coldwell M., Bottley A., Bushell M., Willis A.E., Siddle K. (2009). The human insulin receptor mRNA contains a functional internal ribosome entry segment. Nucleic Acids Res..

[79] Paul S., Dansithong W., Kim D., Rossi J., Webster N.J., Comai L., Reddy S. (2006). Interaction of muscleblind, CUG-BP1 and hnRNP H proteins in DM1-associated aberrant IR splicing. EMBO J..

[80] Talukdar I., Sen S., Urbano R., Thompson J., Yates J.R., Webster N.J. (2011). hnRNP A1 and hnRNP F modulate the alternative splicing of exon 11 of the insulin receptor gene. PLoS ONE.

[81] Cui H., Wu F., Sun Y., Fan G., Wang Q. (2010). Up-regulation and subcellular localization of hnRNP A2/B1 in the development of hepatocellular carcinoma. BMC Cancer.

[82] Sen S., Langiewicz M., Jumaa H., Webster N.J. (2015). Deletion of serine/arginine-rich splicing factor 3 in hepatocytes predisposes to hepatocellular carcinoma in mice. Hepatology.

[83] Xiao M., Lou C., Xiao H., Yang Y., Cai X., Li C., Jia S., Huang Y. (2018). Mir-128 regulation of glucose metabolism and cell proliferation in triple-negative breast cancer. Br. J. Surg..

[84] Lovat F., Fassan M., Gasparini P., Rizzotto L., Cascione L., Pizzi M., Vicentini C., Balatti V., Palmieri D., Costinean S. (2015). miR-15b/16-2 deletion promotes B-cell malignancies. Proc. Natl. Acad. Sci. USA.

[85] Yoshino H., Enokida H., Chiyomaru T., Tatarano S., Hidaka H., Yamasaki T., Gotannda T., Tachiwada T., Nohata N., Yamane T. (2012). Tumor suppressive microRNA-1 mediated novel apoptosis pathways through direct inhibition of splicing factor serine/arginine-rich 9 (SRSF9/SRp30c) in bladder cancer. Biochem. Biophys. Res. Commun..

[86] Wahl M.C., Will C.L., Luhrmann R. (2009). The spliceosome: Design principles of a dynamic RNP machine. Cell.

[87] Will C.L., Luhrmann R. (2011). Spliceosome structure and function. Cold Spring Harb. Perspect. Biol..

[88] Pan Q., Shai O., Lee L.J., Frey B.J., Blencowe B.J. (2008). Deep surveying of alternative splicing complexity in the human transcriptome by high-throughput sequencing. Nat. Genet..

[89] Wang E.T., Sandberg R., Luo S., Khrebtukova I., Zhang L., Mayr C., Kingsmore S.F., Schroth G.P., Burge C.B. (2008). Alternative isoform regulation in human tissue transcriptomes. Nature.

[90] Black D.L. (2000). Protein diversity from alternative splicing: A challenge for bioinformatics and post-genome biology. Cell.

[91] Sen S., Talukdar I., Webster N.J. (2009). SRp20 and CUG-BP1 modulate insulin receptor exon 11 alternative splicing. Mol. Cell. Biol..

[92] Kaminska D., Hamalainen M., Cederberg H., Kakela P., Venesmaa S., Miettinen P., Ilves I., Herzig K.H., Kolehmainen M., Karhunen L. (2014). Adipose tissue INSR splicing in humans associates with fasting insulin level and is regulated by weight loss. Diabetologia.

[93] Chang E.T., Donahue J.M., Xiao L., Cui Y., Rao J.N., Turner D.J., Twaddell W.S., Wang J.Y., Battafarano R.J. (2012). The RNA-binding protein CUG-BP1 increases survivin expression in oesophageal cancer cells through enhanced mRNA stability. Biochem. J..

[94] Philips A.V., Timchenko L.T., Cooper T.A. (1998). Disruption of splicing regulated by a CUG-binding protein in myotonic dystrophy. Science.

[95] Lee E.K., Kim H.H., Kuwano Y., Abdelmohsen K., Srikantan S., Subaran S.S., Gleichmann M., Mughal M.R., Martindale J.L., Yang X. (2010). hnRNP C promotes APP translation by competing with FMRP for APP mRNA recruitment to P bodies. Nat. Struct. Mol. Biol..

[96] Shetty S. (2005). Regulation of urokinase receptor mRNA stability by hnRNP C in lung epithelial cells. Mol. Cell. Biochem..

[97] Paul S., Dansithong W., Jog S.P., Holt I., Mittal S., Brook J.D., Morris G.E., Comai L., Reddy S. (2011). Expanded CUG repeats dysregulate RNA splicing by altering the stoichiometry of the muscleblind 1 complex. J. Biol. Chem..

[98] Shepard P.J., Hertel K.J. (2009). The SR protein family. Genome Biol..

[99] Konieczny P., Stepniak-Konieczna E., Sobczak K. (2014). MBNL proteins and their target RNAs, interaction and splicing regulation. Nucleic Acids Res..

[100] Lin X., Miller J.W., Mankodi A., Kanadia R.N., Yuan Y., Moxley R.T., Swanson M.S., Thornton C.A. (2006). Failure of MBNL1-dependent post-natal splicing transitions in myotonic dystrophy. Hum. Mol. Genet..

[101] Ho T.H., Charlet B.N., Poulos M.G., Singh G., Swanson M.S., Cooper T.A. (2004). Muscleblind proteins regulate alternative splicing. EMBO J..

[102] Sen S., Talukdar I., Liu Y., Tam J., Reddy S., Webster N.J. (2010). Muscleblind-like 1 (MBNL1) promotes insulin receptor exon 11 inclusion via binding to a downstream evolutionarily conserved intronic enhancer. J. Biol. Chem..

[103] Dansithong W., Paul S., Comai L., Reddy S. (2005). MBNL1 is the primary determinant of focus formation and aberrant insulin receptor splicing in DM1. J. Biol. Chem..

[104] Bartel D.P. (2009). MicroRNAs: Target recognition and regulatory functions. Cell.

[105] Yoon J.H., Her S., Kim M., Jang I.S., Park J. (2012). The expression of damage-regulated autophagy modulator 2 (DRAM2) contributes to autophagy induction. Mol. Biol. Rep..

[106] Bartel D.P. (2004). Micrornas: Genomics, biogenesis, mechanism, and function. Cell.

[107] Calin G.A., Croce C.M. (2006). MicroRNA signatures in human cancers. Nat. Rev. Cancer.

[108] Esquela-Kerscher A., Slack F.J. (2006). Oncomirs—MicroRNAs with a role in cancer. Nat. Rev. Cancer.

[109] Suzuki H., Maruyama R., Yamamoto E., Kai M. (2012). DNA methylation and microRNA dysregulation in cancer. Mol. Oncol..

[110] Tsugane S., Inoue M. (2010). Insulin resistance and cancer: Epidemiological evidence. Cancer Sci..

[111] Venables J.P. (2006). Unbalanced alternative splicing and its significance in cancer. Bioessays.

[112] Yoon J.H., Abdelmohsen K., Gorospe M. (2013). Posttranscriptional gene regulation by long noncoding RNA. J. Mol. Biol..

[113] Sisino G., Zhou A.X., Dahr N., Sabirsh A., Soundarapandian M.M., Perera R., Larsson-Lekholm E., Magnone M.C., Althage M., Tyrberg B. (2017). Long noncoding RNAs are dynamically regulated during beta-cell mass expansion in mouse pregnancy and control beta-cell proliferation in vitro. PLoS ONE.

[114] Ellis B.C., Graham L.D., Molloy P.L. (2014). CRNDE, a long non-coding RNA responsive to insulin/IGF signaling, regulates genes involved in central metabolism. Biochim. Biophys. Acta.

[115] Marr M.T., D’Alessio J.A., Puig O., Tjian R. (2007). IRES-mediated functional coupling of transcription and translation amplifies insulin receptor feedback. Genes Dev..

[116] Olson C.M., Donovan M.R., Spellberg M.J., Marr M.T. (2013). The insulin receptor cellular IRES confers resistance to eIF4A inhibition. eLife.

[117] van der Velden A.W., Thomas A.A. (1999). The role of the 5’ untranslated region of an mRNA in translation regulation during development. Int. J. Biochem. Cell Biol..

[118] Gavin J.R., Roth J., Neville D.M., de Meyts P., Buell D.N. (1974). Insulin-dependent regulation of insulin receptor concentrations: A direct demonstration in cell culture. Proc. Natl. Acad. Sci. USA.

[119] Nagarajan A., Petersen M.C., Nasiri A.R., Butrico G., Fung A., Ruan H.B., Kursawe R., Caprio S., Thibodeau J., Bourgeois-Daigneault M.C. (2016). MARCH1 regulates insulin sensitivity by controlling cell surface insulin receptor levels. Nat. Commun..

[120] Zhao T., Xu Y. (2010). P53 and stem cells: New developments and new concerns. Trends Cell Biol..

[121] Zelenko Z., Gallagher E.J., Antoniou I.M., Sachdev D., Nayak A., Yee D., LeRoith D. (2016). EMT reversal in human cancer cells after IR knockdown in hyperinsulinemic mice. Endocr. Relat. Cancer.

[122] Giani F., Vella V., Nicolosi M.L., Fierabracci A., Lotta S., Malaguarnera R., Belfiore A., Vigneri R., Frasca F. (2015). Thyrospheres from normal or malignant thyroid tissue have different biological, functional, and genetic features. J. Clin. Endocrinol. Metab..

[123] Malaguarnera R., Nicolosi M.L., Sacco A., Morcavallo A., Vella V., Voci C., Spatuzza M., Xu S.Q., Iozzo R.V., Vigneri R. (2015). Novel cross talk between IGF-IR and DDR1 regulates IGF-IR trafficking, signaling and biological responses. Oncotarget.

[124] Mata R., Palladino C., Nicolosi M.L., Lo Presti A.R., Malaguarnera R., Ragusa M., Sciortino D., Morrione A., Maggiolini M., Vella V. (2016). IGF-I induces upregulation of DDR1 collagen receptor in breast cancer cells by suppressing MIR-199a-5p through the PI3K/AKT pathway. Oncotarget.

[125] Belfiore A., Malaguarnera R., Nicolosi M.L., Lappano R., Ragusa M., Morrione A., Vella V. (2018). A novel functional crosstalk between DDR1 and the IGF axis and its relevance for breast cancer. Cell Adh. Migr..

[126] Vella V., Malaguarnera R., Nicolosi M.L., Palladino C., Spoleti C., Massimino M., Vigneri P., Purrello M., Ragusa M., Morrione A. (2017). Discoidin domain receptor 1 modulates insulin receptor signaling and biological responses in breast cancer cells. Oncotarget.

[127] Vella V., Nicolosi M.L., Cantafio P., Massimino M., Lappano R., Vigneri P., Ciuni R., Gangemi P., Morrione A., Malaguarnera R. (2018). DDR1 regulates thyroid cancer cell differentiation via IGF-2/IR-A autocrine signaling loop. Endocr. Relat. Cancer.

[128] Cao H., Dong W., Shen H., Xu J., Zhu L., Liu Q., Du J. (2015). Combinational therapy enhances the effects of anti-IGF-1R mAb Figitumumab to target small cell lung cancer. PLoS ONE.

[129] King H., Aleksic T., Haluska P., Macaulay V.M. (2014). Can we unlock the potential of IGF-1R inhibition in cancer therapy?. Cancer Treat. Rev..

[130] Livingstone C. (2013). IGF2 and cancer. Endocr. Relat. Cancer.

[131] Malaguarnera R., Vella V., Nicolosi M.L., Belfiore A. (2017). Insulin resistance: Any role in the changing epidemiology of thyroid cancer?. Front. Endocrinol. (Lausanne).

[132] Sciacca L., Vella V., Frittitta L., Tumminia A., Manzella L., Squatrito S., Belfiore A., Vigneri R. (2018). Long-acting insulin analogs and cancer. Nutr. Metab. Cardiovasc. Dis..

[133] Buck E., Gokhale P.C., Koujak S., Brown E., Eyzaguirre A., Tao N., Rosenfeld-Franklin M., Lerner L., Chiu M.I., Wild R. (2010). Compensatory insulin receptor (IR) activation on inhibition of insulin-like growth factor-1 receptor (IGF-1R): Rationale for cotargeting IGF-1R and IR in cancer. Mol. Cancer Ther..

[134] Ulanet D.B., Ludwig D.L., Kahn C.R., Hanahan D. (2010). Insulin receptor functionally enhances multistage tumor progression and conveys intrinsic resistance to IGF-1R targeted therapy. Proc. Natl. Acad. Sci. USA.

[135] Dinchuk J.E., Cao C., Huang F., Reeves K.A., Wang J., Myers F., Cantor G.H., Zhou X., Attar R.M., Gottardis M. (2010). Insulin receptor (IR) pathway hyperactivity in IGF-IR null cells and suppression of downstream growth signaling using the dual IGF-IR/IR inhibitor, BMS-754807. Endocrinology.

[136] Garofalo C., Manara M.C., Nicoletti G., Marino M.T., Lollini P.L., Astolfi A., Pandini G., Lopez-Guerrero J.A., Schaefer K.L., Belfiore A. (2011). Efficacy of and resistance to anti-IGF-1R therapies in ewing’s sarcoma is dependent on insulin receptor signaling. Oncogene.

[137] Zhang H., Pelzer A.M., Kiang D.T., Yee D. (2007). Down-regulation of type I insulin-like growth factor receptor increases sensitivity of breast cancer cells to insulin. Cancer Res..

[138] Mulvihill M.J., Cooke A., Rosenfeld-Franklin M., Buck E., Foreman K., Landfair D., O’Connor M., Pirritt C., Sun Y., Yao Y. (2009). Discovery of OSI-906: A selective and orally efficacious dual inhibitor of the IGF-1 receptor and insulin receptor. Future Med. Chem..

[139] Kheirandish M., Mahboobi H., Yazdanparast M., Kamal W., Kamal M.A. (2018). Anti-cancer effects of metformin: Recent evidences for its role in prevention and treatment of cancer. Curr. Drug Metab..

[140] Vella V., Nicolosi M.L., Giuliano S., Bellomo M., Belfiore A., Malaguarnera R. (2017). PPAR-gamma agonists as antineoplastic agents in cancers with dysregulated IGF axis. Front. Endocrinol. (Lausanne).

[141] Wang Q., Ru Y., Zhong D., Zhang J., Yao L., Li X. (2014). Engineered ubiquitin ligase PTB-U-box targets insulin/insulin-like growth factor receptor for degradation and coordinately inhibits cancer malignancy. Oncotarget.

